# A Fatal Case of Cerebral Fat Embolism: A Case Report

**DOI:** 10.7759/cureus.59107

**Published:** 2024-04-26

**Authors:** Xi Yao Gui, Waqas Ahmad, Ismail Ali

**Affiliations:** 1 Department of Radiology, University of British Columbia, Faculty of Medicine, Vancouver, CAN; 2 Department of Radiology, Vancouver General Hospital, Vancouver, CAN

**Keywords:** computed tomography, magnetic resonance imaging, brain, fracture, fat embolism

## Abstract

Fat embolism syndrome (FES) is a rare but serious multisystem syndrome that occurs after 0.9% to 2.2% of fractures, with long bone and pelvic fractures being the most common. The classic triad of FES consists of neurological impairment, respiratory insufficiency, and petechial rash, which develops 12-72 hours after the initial incident. We hereby present a case of a patient who developed persistent altered consciousness, seizures, and hypoxia secondary to a comminuted sacral fracture. Although the patient could not survive owing to multiple factors, imaging played a pivotal role in expediting the diagnostic process and aiding early management.

## Introduction

Fat embolism syndrome (FES) is a rare but serious multisystem syndrome that occurs after 0.9 to 2.2% of fractures, with long bone and pelvic fractures being the most common [1,2].

One theory of the pathogenesis of FES posits that elevated intramedullary pressure after orthopedic trauma leads to bone marrow entering damaged venous sinusoids and the liberation of fat droplets into the venous circulation. These fat droplets can obstruct flow within small vessels, such as the pulmonary capillaries. Subsequently, either via an arteriovenous shunt, such as a patent foramen ovale, or directly through the pulmonary capillary bed, these fat droplets can cause arterial occlusions [3].

The classic triad of FES consists of neurological impairment, respiratory insufficiency, and petechial rash, which develops 12-72 hours after the initial incident [3]. There is no gold standard for the diagnosis of FES. A frequently used clinical diagnosis tool is Gurd’s criteria, but it may not be satisfied in all patients. In Gurd’s criteria, at least two major criteria or one major and four minor criteria must be satisfied. The major criteria are cerebral involvement, respiratory insufficiency, and petechial rash. Minor criteria are tachycardia, fever, retinal changes, jaundice, renal signs, thrombocytopenia, anemia, high ESR, and fat macroglobulinemia [4]. The brain and lungs are the most commonly affected sites, and between 33% and 86% of patients exhibit neurological manifestations either alongside our patient or following pulmonary manifestations [3].

## Case presentation

**Figure 1:**
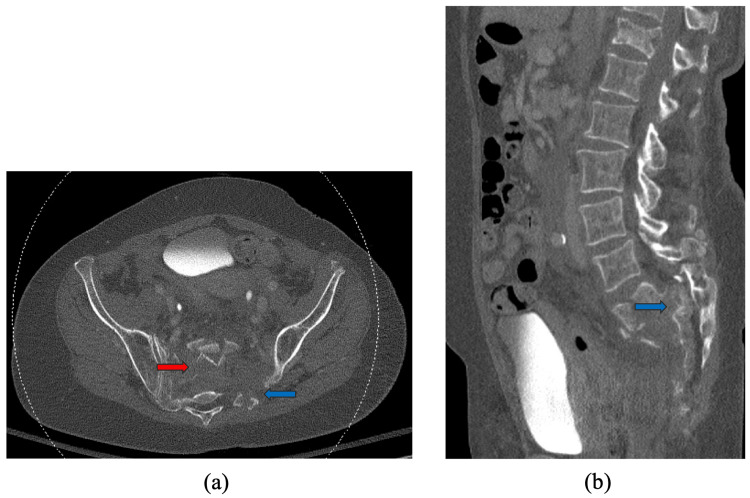
Axial (a) and sagittal (b) bone window images of the pelvis showing comminuted fracture of the sacrum with intra-articular extension (red and blue arrows), extension into the bilateral S1 and S2 neural foramina (blue arrows), and obliteration of the sacral spinal canal (blue arrows).

A 69-year-old previously healthy female presented to the hospital after a fall from 10 m. At the scene, her Glasgow Coma Scale (GCS) was seven, which improved to 15 upon arrival at the hospital. Initial CT of the pelvis demonstrated a comminuted fracture of the sacrum with intra-articular extension, extension into the bilateral S1 and S2 neural foramina, and obliteration of the sacral spinal canal (Figure 1). The initial CT of the head was normal.

**Figure 2:**
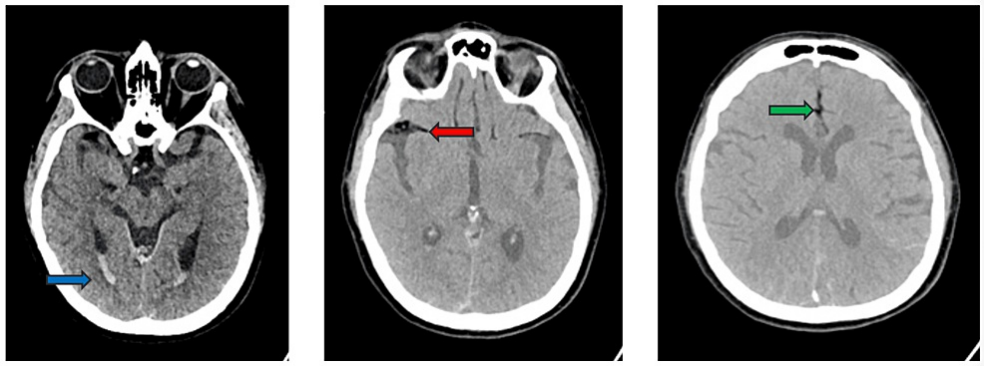
Axial CT images of the brain showing intraventricular bleed (blue arrow), as well as fat density material within the anterior aspect of the right sylvian fissure (red arrow) and within the anterior interhemispheric fissure (green arrow).

**Figure 3:**
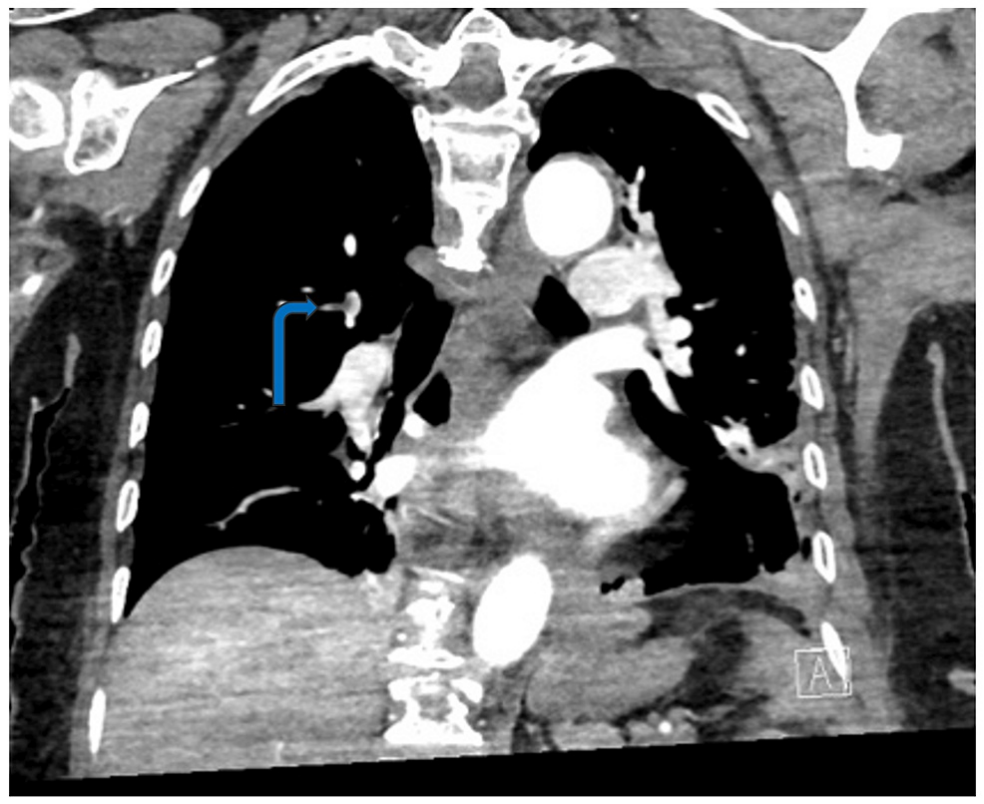
Coronal CT pulmonary angiogram image showing right upper lobe fat embolus extending from segmental to subsegmental branch (arrow).

Twenty-nine hours after the incident, she underwent emergency posterior L4 to L5 and sacroiliac fusion surgery and was under general anesthesia for five hours. Post-operatively, at 40 hours after the incident, her GCS dropped to five, and her oxygen requirement increased to 4 L/min. There was no petechial rash. A CT of the head done at this stage demonstrated subarachnoid hemorrhage with minimal intraventricular hemorrhage. The note was made of fat-density material within the subarachnoid space in the anterior aspect of the right Sylvian fissure in the region of the middle cerebral artery bifurcation, anteriorly along the bilateral temporal lobes, in the suprasellar region, and within the anterior interhemispheric fissure (Figure 2). An MRI was suggested for further assessment. A concurrent CT angiogram of the chest demonstrated fat-density pulmonary emboli in the distal right upper lobe posterior segmental pulmonary artery extending into the subsegmental branches (Figure 3).

**Figure 4:**
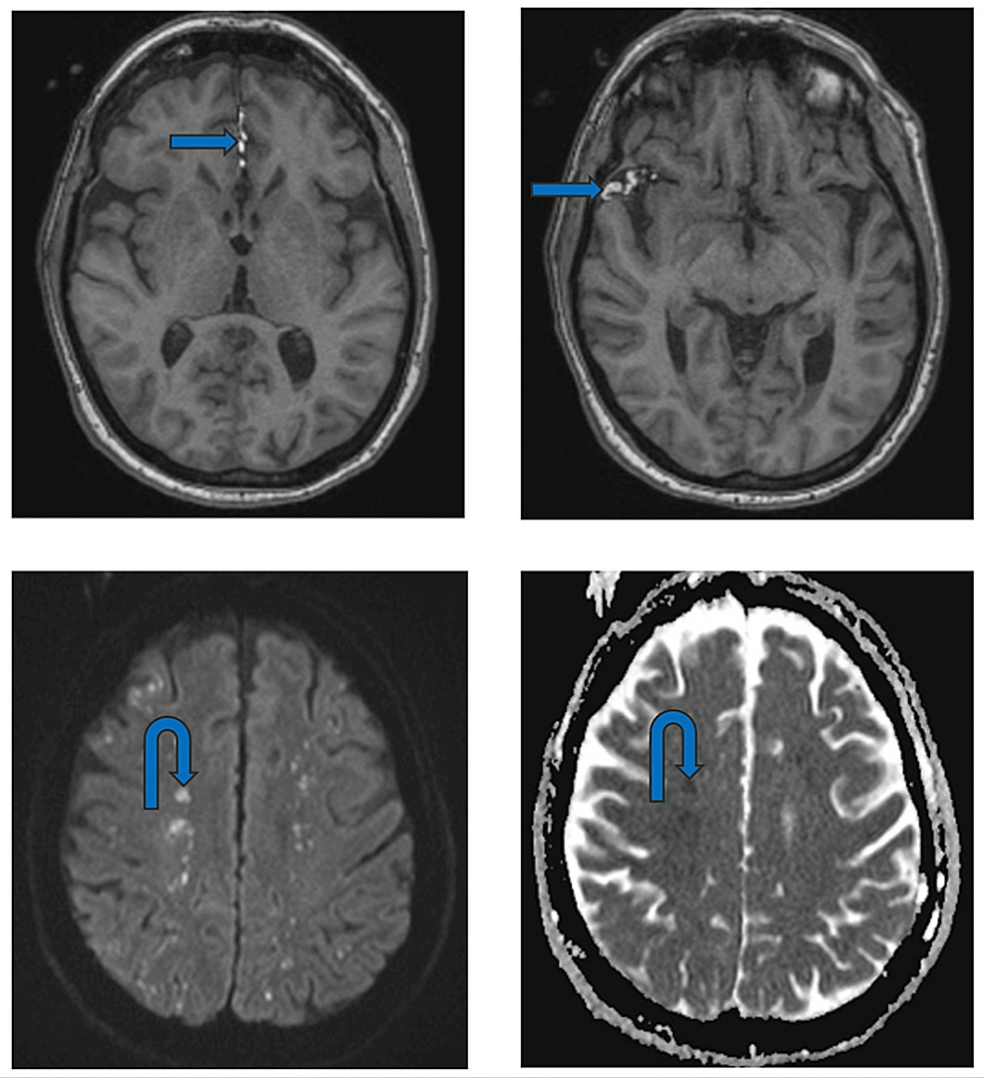
Axial MRI T1 images of the brain showing high signal fat in the sylvian fissure and interhemispheric area (arrows). DWI and ADC images showing changes in watershed regions (curved arrows).

An MRI of the brain showed T1 high-fat signal changes, confirming CT findings, likely tracking from the pelvic fracture via a dural tear. Diffusion imaging demonstrated supratentorial and infratentorial foci of restricted diffusion and susceptibility, predominantly in a watershed territory distribution (Figure 4). Given the MRI findings, clinical history, and extensive pelvic fracture, the most likely diagnosis was cerebral FES.

Seventy-five hours after the incident, she developed minimal convulsive status epilepticus, which was demonstrated on an electroencephalogram, and she was started on antiepileptic therapy. An echocardiogram demonstrated a patent foramen ovale. The patient did not survive due to her persistently limited neurological function, poor prognosis, and extent of injuries.

## Discussion

There are often challenges with diagnosing FES clinically, which lie in its variable time of onset, multifaceted presentation, and different degrees of severity [4]. Neurological signs and symptoms, in particular, can be variable and nonspecific [3]. Neuroimaging can expedite the diagnostic process and eliminate the need for other unnecessary investigations and interventions [3]. In addition, neuroimaging is useful for prognosis. Given our patient’s nonspecific neurological presentation, acute stroke, diffuse axonal injury (DAI), and effects of prolonged anesthesia during surgery were possible clinical differentials; however, given her extensive pelvic fracture, a high index of suspicion was maintained for FES, and additional neurological and pulmonary imaging was ordered.

In pulmonary FES, chest radiographs may be normal in mild cases [5]. However, the patchy or diffuse bilateral opacities that are sometimes found are non-specific and can resemble findings in acute respiratory distress syndrome from any cause [5,6]. Interstitial and nodular opacities may also be found. CT is superior and may demonstrate areas of consolidation, ground-glass opacities, and small (2 to 10 mm) nodules [5]. These nodules could represent alveolar edema, inflamed intrapulmonary lymph nodes, or hemorrhage secondary to the FES [7-9]. Findings of fat-attenuating filling detected in the pulmonary arteries from pulmonary FES are infrequent [9].

In acute neurological dysfunction, CT of the head is frequently the first imaging modality performed due to its rapidity and accessibility [1]. During the acute phase, CT may occasionally delineate edematous areas with low attenuation and hemorrhagic areas with high attenuation [10,11]. However, it is often unremarkable in cerebral FES. Therefore, magnetic resonance imaging is the recommended modality for patients with clinically suspected cerebral FES [11]. Cerebral FES is an evolving process that presents distinct imaging patterns at different time windows. When there is clinical suspicion of cerebral FES, diffusion-weighted imaging (DWI) and susceptibility-weighted imaging (SWI) should be performed early to expedite the diagnostic process. DWI and SWI are more sensitive than fluid-attenuated inversion recovery and T2-weighted imaging (T2WI) for the early detection of characteristic patterns of cerebral FES [12].

During the acute stage, most common at one to four days, the “starfield pattern” is the most observed. It is identified on DWI as areas with restricted diffusion and on T2WI as iso- or hyperintense areas [12]. The “starfield pattern” is defined by numerous dispersed, small, and hyperintense lesions against a dark background that is found in both white and deep gray matter, especially along the boundary zones of major vascular territories [13,14]. While the “starfield pattern” is the most recognized manifestation of cerebral FES, it is non-specific and can be seen in various types of embolic events. However, in the context of cerebral FES, these lesions are often transient [12]. The “starfield pattern” corresponds to low-value lesions on the apparent diffusion coefficient (ADC) map, which is consistent with restricted diffusion and is indicative of acute ischemia and cytotoxic edema [13].

During the subacute stage, most common at five to 14 days, two distinct patterns can be observed. The first pattern is characterized by confluent cytotoxic edema in the white matter. On DWI, this is denoted by confluent symmetric lesions with restricted diffusion, particularly in the bilateral periventricular and subcortical white matter, as well as the cerebellar peduncles, corpus callosum, and posterior internal capsule. On T2WI, the lesions appear either slightly hyperintense or have no signal change. The second pattern is characterized by vasogenic edema lesions with possible enhancement. These lesions are normally small and patch-shaped and are present in both white and gray matter. They demonstrate increased diffusion on DWI and hyperintensity on T2WI. Additionally, they may exhibit enhancement following contrast administration [12].

SWI facilitates the identification of petechial hemorrhages, which is another hallmark feature of cerebral FES that can be observed in all stages. These profuse microhemorrhages are seen as a “walnut kernel pattern” [12]. These lesions reside in the perivascular space at both the white and deep gray matter levels in the cerebellum, brainstem, and corpus callosum. It is important to differentiate these lesions from petechial hemorrhages caused by DAI, which is another potential outcome after trauma. On T2WI, cerebral FES lesions typically appear more numerous and widespread than those from DAI, which tends to exhibit larger and more linear hemorrhages. Moreover, confluent diffusion restriction on DWI is typical of cerebral FES, while a few scattered foci are typical of DAI [15].

At the late stage, most of the abovementioned lesions may lead to cerebral atrophy. In addition, the sequelae of infarction, gliosis, and chronic demyelination may manifest as increased diffusion on DWI and hyperintense dot-shaped lesions on T2W1 [12].

Cerebral FES is usually self-limiting and completely resolves within a few weeks to years [3]. However, there is still a mortality rate of up to 10% [16]. As previously mentioned, the “starfield pattern” observed on DWI is attributed to fat droplets that have bypassed the pulmonary filter and reached the brain’s capillaries to cause ischemia [12]. These lesions tend to be reversible since fat droplets can deform and recycle into the pulmonary circulation [17,18]. This explains why cerebral FES has a more favorable outcome than other types of embolic events [3]. However, if there is no pulmonary filter, such as when a patent foramen ovale is present, there tends to be earlier presentations of neurological signs and symptoms, larger brain lesions, more territorial infarctions, and worse clinical prognoses [3]. Our patient’s clinical course was complicated by the severity of her pelvic fracture, a patent foramen ovale, and her advanced age.

Early diagnosis and treatment of cerebral FES reduces morbidity and mortality [4]. To date, there are no established treatment guidelines, and the primary approach remains supportive therapy [16]. Initiating hyperbaric oxygen therapy early can increase oxygen diffusion into the brain, stimulate capillary growth to foster collateral circulation in the brain, and restore the functionality of brain cells [19].

## Conclusions

FES is a rare but serious multisystem syndrome. For patients with complex fractures and neurological deterioration, clinical suspicion should be high for cerebral FES despite a normal initial head CT. While FES is usually self-limiting, it can become fatal with delayed diagnosis and management. There are often challenges with diagnosing FES clinically. Neuroimaging can be used to expedite the diagnostic process, and MRI is the recommended modality. Specifically, DWI and SWI are more sensitive than fluid-attenuated inversion recovery and T2WI for the early detection of characteristic patterns of cerebral FES.
